# HEA-Net: Attention and MLP Hybrid Encoder Architecture for Medical Image Segmentation

**DOI:** 10.3390/s22187024

**Published:** 2022-09-16

**Authors:** Lijing An, Liejun Wang, Yongming Li

**Affiliations:** College of Information Science and Engineering, Xinjiang University, Urumqi 830000, China

**Keywords:** attention, MLP, Transformer

## Abstract

The model, Transformer, is known to rely on a self-attention mechanism to model distant dependencies, which focuses on modeling the dependencies of the global elements. However, its sensitivity to the local details of the foreground information is not significant. Local detail features help to identify the blurred boundaries in medical images more accurately. In order to make up for the defects of Transformer and capture more abundant local information, this paper proposes an attention and MLP hybrid-encoder architecture combining the Efficient Attention Module (EAM) with a Dual-channel Shift MLP module (DS-MLP), called HEA-Net. Specifically, we effectively connect the convolution block with Transformer through EAM to enhance the foreground and suppress the invalid background information in medical images. Meanwhile, DS-MLP further enhances the foreground information via channel and spatial shift operations. Extensive experiments on public datasets confirm the excellent performance of our proposed HEA-Net. In particular, on the GlaS and MoNuSeg datasets, the Dice reached 90.56% and 80.80%, respectively, and the IoU reached 83.62% and 68.26%, respectively.

## 1. Introduction

Medical image segmentation is a fundamental and critical task in computer-aided diagnosis, which aims to accurately identify the target organ, tissue, or lesion area at the pixel level. As opposed to images of natural scenes, medical images are often complicated in texture and limited by imaging technology and equipment. Although the research [1,2] on artificial intelligence-based enhanced magnetic resonance imaging has made contributions to medical image processing, medical images are still characterized by noise interference, blurring, and are difficult to judge. Moreover, medical image labeling intensively depends on the cognition and experience of the medical experts, which results in significant labeling errors and causes there to be few annotated data records available for training. From the characteristics mentioned above of blurred edges, less training data, and inevitable labeling errors in medical images, the auxiliary diagnosis system based on traditional image segmentation algorithms is not enough to meet the requirements of clinical applications.

In recent years, CNN has gradually become a mainstream image-processing method. Subsequently, researchers proposed a full convolutional neural network (FCN) [3] and U-Net [4] for image segmentation, with the latter being more widely used. Due to its remarkable performance in image segmentation, U-Net is used in the segmentation of the eye [5], heart [6,7], liver, brain [8], skin [9], prostate [10], breast, and other organs. Relying on U-Net, researchers have made great strides in practical application and network performance, such as ANU-Net [11], DIU-Net [12], AttUNet [13], MRUNet [14], HDC-Net [15], and FAC-Net [16]. Although U-Net [4] and its derived models have made great achievements in the field of medical image segmentation, they all face the inevitable problem similar to CNN models: a lack of long-term global correlation modeling capability. The main reason is that CNN simply extracts local information and cannot effectively measure global correlation.

Much recent work has attempted to address this problem by using Transformer. Transformer is a model based on self-attention (SA). SA’s ability to model the dependencies between all of the input elements means that Transformer can handle global long-term dependencies. Some recent work has yielded satisfactory results, such as that of Chen et al., who first proposed the Transformer architecture TransUNet [17] for medical image segmentation. It uses CNN to extract features and inputs the extracted features into Transformer for long-range dependency computation. Zhang et al. proposed TransFuse [18] based on ViT [19], which integrates features extracted from Transformer and CNN. Valanarasu et al. proposed MedT [20] based on Axis-attention [21], which proved that Transformer could be applied to small datasets. Since it is difficult to distinguish the boundary of the foreground area of medical images, the local detail information is also important to the segmentation result. However, Transformer focuses on extracting global information while weakening local information, so it also has some shortcomings in the task of medical image segmentation. How to properly strengthen foreground information, suppress useless background information, and how to better attain the joint modeling of local and global features have become the focus of our research.

To solve the above problems, we designed a new attention mechanism, EAM, and proposed DS-MLP. We used EAM to effectively connect the convolution block to Transformer. EAM attention enhances the model’s perception of the boundary of the sample foreground region and weakens the background information, thus providing richer foreground information for the global modeling of Transformer. We further propose DS-MLP to strengthen the acquired features and obtain more significant coding ability for our model.

We summarize the contributions of this paper as follows:

(1) We propose an Efficient Attention Module (EAM) that enhances the foreground and suppresses invalid background information in medical images by performing feature refinement at the channel and spatial level;
(2) We propose a Dual-channel Shifted MLP module (DS-MLP), which can further enhance foreground information via channel and spatial shift operations.

We briefly review the related network models in Section 2. The third part introduces the general framework of our network and the details of associated modules. The fourth part explicates the datasets and indicators used. The fifth part presents our experimental results and experimental analysis. The sixth part is the conclusion.

## 2. Related Work

CNN uses convolution as a primary operator to collect the local features and filter images in a sliding window. Typical networks in image segmentation are encoder-decoder-based models, such as R2U-net [22], U-Net [4], DenseUNet [23], UNet++ [24], ResUNet++ [25], etc. They have gradually dominated the field with their excellent performance. However, the local features collected by convolution alone are not enough to distinguish the foreground information from background information in medical images.

In recent years, the attention mechanism has been proved to be a potential means of enhancing deep CNN. Since then, researchers have proposed a variety of attention modules to strengthen the capability of a convolutional neural network. For example, SENet [26] can adaptively adjust the feature response values of each channel and model the internal dependence between the channels. BAM [27] infers attention maps along two different paths (channels and spaces) and embeds them in each bottleneck block. CBAM [28] provides a solution to embed channel and spatial attention submodules sequentially. To avoid the neglect of cross-dimensional interaction, TAM [29] considered dimensional correlation by rotating the feature mapping. However, existing attention modules are composed of multiple layers of convolution or pooling, which inevitably increases the complexity of the module.

Natural Language Processing (NLP) was the first field to apply the Transformer architecture. Researchers applied Transformer to computer vision joining the popularity of Transformer with NLP applications. Most of the networks used for medical image segmentation are based on hybrid architectures of Transformer and CNN. For example, Gao et al. proposed UTNet [30], which applied self-attention modules to the encoder and decoder to obtain long-term dependence relations of different scales at the lowest cost. The UCTransNet [31] proposed by Wang et al. utilized the CTrans module as a skip connection to solve the problem of the semantic gap. There are also architectures based on pure Transformer, such as Swin-Unet [32] proposed by Cao et al., where each module of the network is built based on the Swin Transformer block [33].

CNN can only simply extract local information, but cannot effectively measure global relevance. Transformer mainly models the global context of an image, which has limited awareness of local features. Since it is difficult to distinguish the boundary of the foreground area of a medical image, the local detail information is also important to the segmentation result. Based on this, we propose HEA-Net. In our approach, we make use of the attention mechanism to effectively connect the convolution blocks with Transformer to form a powerful, joint local and global model.

## 3. Methods

**Figure 1:**
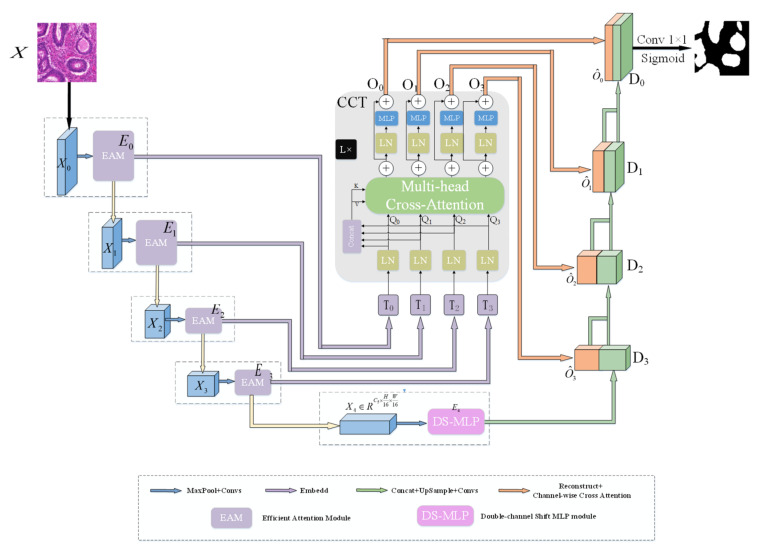
The architecture diagram of HEA-Net.

The overall architecture of the network is shown in Figure 1. The network consists of four parts: encoder, skip connection, bottleneck block, and decoder. The encoder uses EAM and convolution layers for feature extraction. As shown in Figure 1, EAM enhances the foreground and suppresses the invalid background information in medical images by performing feature refinement at the channel and spatial levels. In order to provide the decoder with more refined foreground information, DS-MLP further enhances the foreground information via the channel and spatial shift operations and embeds it into the bottleneck block. The skip connections and decoders are aligned with UCTransNet [31].

### 3.1. Efficient Attention Module (EAM)

Medical research in recent years has shown that the early treatment of most diseases largely depends on the quality of lesion segmentation. However, the exceptionally high similarity between the diseased tissue and the surrounding tissue makes the edges blurred and not easily recognized by the naked eye. This section introduces the EAM module, which can adequately detect and segment the diseased tissue margins and nuclear boundaries.

**Figure 2:**
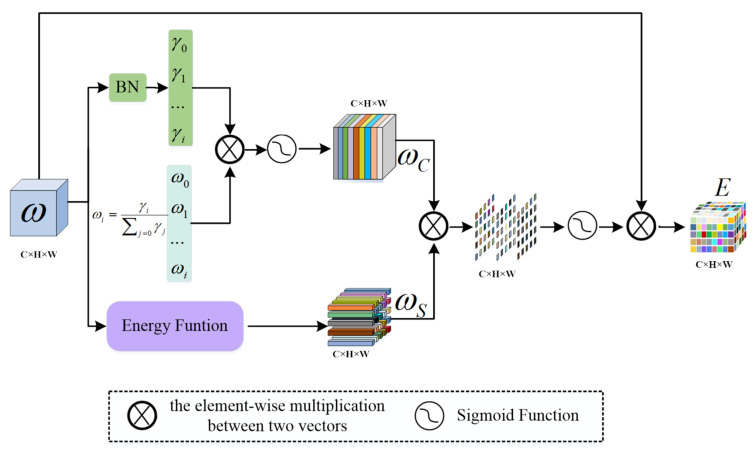
Efficient Attention Module.

As shown in Figure 2, the EAM module inputs feature maps into the two branches to estimate the weights of channel dimension and spatial dimension, respectively. In terms of channel dimension, the scale factor (γ) in batch normalization (*BN*) [34] assigns a unique weight to each channel in the input feature map to extract different feature information from each channel. The weights are calculated as follows [35]:

(1) $$\omega_{\gamma }=\frac{\gamma_{i}}{\sum_{j=0}\gamma_{j}},$$

where γ is the scale factor; i and j are indexes over the channel dimension. Therefore, the calculation formula for the channel dimension is as follows:

(2) $$\omega_{C}=sigmoid\left(\omega_{\gamma }⊗BN\left(\omega \right)\right),$$

where ⊗ denotes the element-wise multiplication; BN denotes batch normalization; and $\omega \in R^{C\times H\times W}$ represents the input image.

In the spatial dimension, the minimum energy function [36] within a single channel is used to estimate the weight of each neuron within the spatial dimension of the image to extract the edge information of the lesion area and nucleus. The minimum energy function [36] was proposed by Yang et al. By summarizing some of the neuroscience findings, they found that the neurons with a large amount of information can show different firing patterns from the surrounding neurons. They define an energy function for each neuron by measuring the linear separability between one target neuron and other neurons. Assuming that all of the pixels on a single channel follow the same distribution, the mean and variance of all neurons can be calculated and reused for all neurons on that channel [37]. Finally, a minimum energy function is obtained:

(3) $$e_{t}^{*}=\frac{4\left({\hat{\sigma }}^{2}+\lambda \right)}{{\left(t-\hat{\mu }\right)}^{2}+2{\hat{\sigma }}^{2}+2\lambda },$$

where $\hat{\mu }=\frac{1}{M}\sum_{i=1}^{M}x_{i}$ and ${\hat{\sigma }}^{2}=\frac{1}{M}{\sum_{i=1}^{M}\left(x_{i}-\hat{\mu }\right)}^{2}$; M=H×W is the number of neurons on that channel; t is the target neuron; and λ is a hyperparameter. The importance of each neuron can be obtained by $\frac{1}{e_{t}^{*}}$. In the experiment, λ is set to 0.0001. Therefore, the calculation of the spatial dimension is as follows:

(4) $$\omega_{S}=\frac{1}{e_{t}^{*}}$$

To obtain the importance of each neuron in the channel dimension and the spatial dimension simultaneously, we combined the weight of the channel dimension with the weight of the spatial dimension by multiplying the elements. The output of the EAM module can be expressed as:

(5) $$E=sigmoid\left(\omega_{C}⊗\omega_{S}\right)⊗\omega ,$$

where ⊗ denotes the element-wise multiplication and $\omega \in R^{C\times H\times W}$ represents the input image.

### 3.2. Dual-Channel Shift MLP Module (DS-MLP)

**Figure 3:**
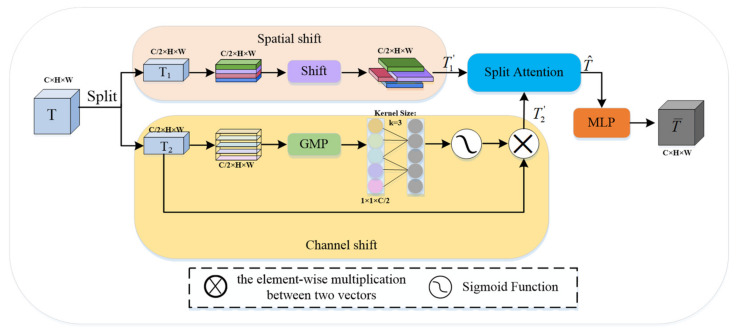
Dual-channel Shift MLP module.

The spatial shift MLP [38] module adopts spatial shift operations to realize the communication between patches. However, the effect of feature enhancement using only spatial shift operation is weak, and Global Maximum Pooling (GMP) can collect more salient features. The DS-MLP module implements further feature enhancement using space shift operations and channel shift operations. The complete structure of the DS-MLP module is shown in Figure 3, and the number of channels and resolution of its output and input feature map remain unchanged.

Given an input feature map $T\in R^{W\times H\times C}$, T is divided into two parts along the channel dimension, represented by $T_{1}$ and $T_{2}$, respectively, $T_{1},T_{2}\in R^{W\times H\times \frac{C}{2}}$:

(6) $$T_{1}=T\left[:,:,1:\frac{C}{2}\right],T_{2}=T\left[:,:,\frac{C}{2}+1:C\right],$$

where C indicates the number of channels.

We carried out two different shift operations for $T_{1}$ and $T_{2}$ to achieve better feature enhancement, namely $SS\left(\cdot \right)$ and $CS\left(\cdot \right)$. $SS\left(\cdot \right)$ performs the same spatial shift operation as [38]. That is, $T_{1}$ is equally divided into four parts along the channel dimension and moved in four directions:

(7) $$T_{11}\left[1:w,:,1:c/4\right]\leftarrow T_{11}\left[0:w-1,:,1:c/4\right],$$

(8) $$T_{12}\left[0:w-1,:,c/4+1:c/2\right]\leftarrow T_{12}\left[1:w,:,c/4+1:c/2\right],$$

(9) $$T_{13}\left[:,1:h,c/2:3c/4\right]\leftarrow T_{13}\left[:,0:h-1,c/2:3c/4\right],$$

(10) $$T_{14}\left[:,0:h-1,3c/4:c\right]\leftarrow T_{14}\left[:,1:h,3c/4:c\right],$$

where w is the width of the feature map; h is the height of the feature map; and c is the number of channels. $CS\left(\cdot \right)$ performs the channel shift operation on $T_{2}$. Specifically, we used GMP to extract dominant features without dimensionality reduction, then used one-dimensional convolution to carry out channel shift operation to realize cross-channel information interaction, and finally, the final channel shift descriptor is generated by sigmoid function and residual connection; the formula is as follows:

(11) $$T_{2}^{\prime }=sigmoid\left(conv\left(GMP\left(T_{2}\right)\right)\right)⊗T_{2},$$

where conv denotes a one-dimensional convolution of kernel size 3; GMP denotes global max pooling; and ⊗ denotes the element-wise multiplication. Then, the two feature maps, $T_{1}$ and $T_{2}$, obtained through different operations are fused using the Split Attention (SA) module [39] to achieve feature enhancement.

**Figure 4:**
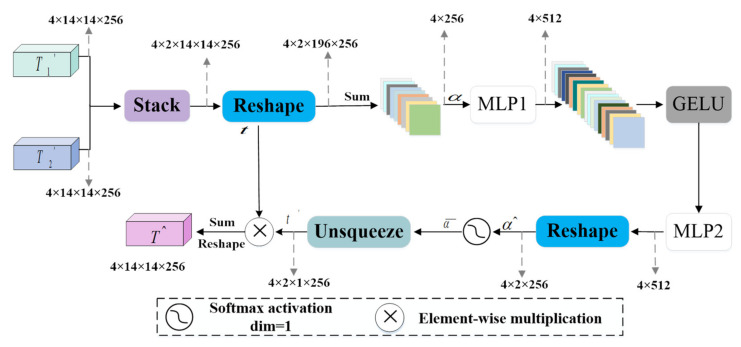
Split Attention module.

In the SA module, $T_{1}^{\prime }$, $T_{2}^{\prime }$ are reshaped into matrices ${\left\{T_{k}\right\}}_{k=1}^{2}$. We used $\left[t_{1},t_{2},\ldots ,t_{S}\right]$ to represent the S feature maps with a size of N×C, where N is the number of patches and C is the number of channels. The average value is taken:

(12) $$\alpha =\sum_{s=1}^{S}1t_{S},$$

where $1\in R^{n}$ is all the n-dimensional row vectors of 1s, $\alpha \in R^{C}$. Generated by a series of MLPs and activation functions:

(13) $$\hat{\alpha }=MLP2\left(GELU\left(MLP1\left(\alpha \right)\right)\right),$$

where $\hat{\alpha }\in R^{C}$. Further processing along one dimension with softmax function the following is obtained:

(14) $$\bar{\alpha }=soft\max\left(\hat{\alpha }\right),$$

where $\bar{\alpha }\in R^{C}$. The workflow of the SA module is shown in Figure 4, and the output of the SA module can be expressed as:

(15) $$\hat{T}=t^{\prime }⊗t,$$

where ⊗ denotes the element-wise multiplication; t is the output of the reshaping operation; $t^{\prime }$ is the output of the un-squeezing operation.

Finally, the output of the DS-MLP module can be expressed as:

(16) $$\bar{T}=MLP\left(\hat{T}\right),$$

where $\hat{T}$ is the output of the SA module.

### 3.3. Channel-Wise Cross Fusion Transformer (CCT)

In the skip connection part, this paper uses the CCT module in UCTransNet (Figure 1) [31]. This module can not only fuse features from different scales in the encoder but also exchange information across channels. The output of the CCT module is:

(17) $$MCA_{i}=\left(CA_{i}^{1}+CA_{i}^{2}+\ldots +CA_{i}^{N}\right)/N,$$

(18) $$O_{i}=MCA_{i}+MLP\left(Q_{i}+MCA_{i}\right),$$

where $CA_{i}$ is a cross-attention (CA); N is the number of heads; $O_{i}$ is the output of the CCT module; $Q_{i}=T_{i}W_{Q_{i}}$, $W_{Q_{i}}\in {\mathbb{R}}^{C_{i}\times d};$ d is the patch numbers; and $C_{i}$ are the channel dimensions of the four EAM modules. Layer normalization (LN) is omitted in Formula (18), and the Lth layer CCT module can be constructed by repeating the formula (18). In the experiment, N and L are both set to 4.

### 3.4. Decoder

**Figure 5:**
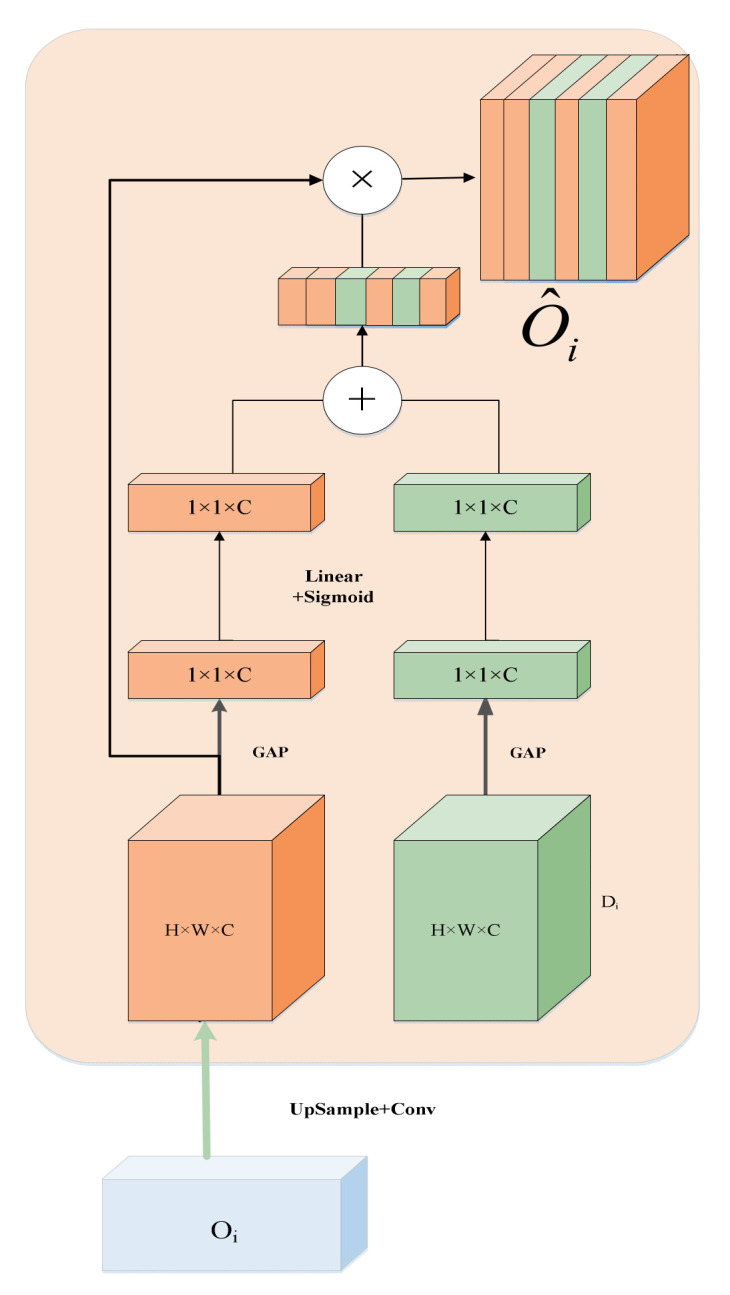
Channel-wise Cross Attention module.

In the decoder, we use the Channel-wise Cross Attention (CCA) module [31] to fuse the semantic features between the output of the CCT module and the upsampling. The CCA module can filter the features generated by the Transformer and reduce the ambiguity of the decoder features. The detailed structure of CCA is shown in Figure 5.

## 4. Experiments

### 4.1. Datasets

GlaS [40] is a glandular segmentation dataset from colon tissue images. There were 165 images in this dataset, including 85 in the training set and 80 in the test set. These images are sections of colorectal adenocarcinoma. The slides were processed on different occasions in the laboratory, and each slide belonged to a different patient. 

MoNuSeg [41] is a multi-organ nuclear segmentation dataset. The training set of this dataset has 30 images with 21,623 independent nuclei annotated. These images contain seven organs: stomach, prostate, liver, colon, bladder, kidney, and breast. The test set of this dataset has 14 images containing the seven organs of the brain, breast, lung, prostate, bladder, colon, and kidney. The lung and brain tissue images are unique to the test set, making testing more challenging.

### 4.2. Evaluation Metrics

The experiment uses the Dice coefficient (Dice) and intersection ratio (IoU) to evaluate network performance. These two indicators are related to four values, namely TP, FP, TN, and FN. TP indicates that the predicted result is the same as the actual result, and both are positive, meaning that the prediction is correct. FP implies that the prediction result is positive, but the actual value is negative, indicating that the model misjudges a negative value as a positive value and the model predicts incorrectly. TN demonstrates that the prediction result is the same as the actual, and both values are negative, indicating that the model predicts correctly. FN demonstrated that the prediction result is negative, but the real value is positive, indicating that the model misjudges a positive value as a negative value, and the model predicts incorrectly. The calculation formulas of the Dice and IoU indicators are as follows:

(19) $$Dice=\frac{2TP}{2TP+FP+FN},$$

(20) $$IoU=\frac{TP}{TP+FP+FN}$$

### 4.3. Implementation Details

We used flipped (vertical and horizontal) and rotated (random) forms of online data augmentation to avoid overfitting. Our proposed HEA-Net is not trained with any pre-trained weights. We set the input resolution size of GlaS to 224 × 224, the input resolution size of MoNuSeg to 512 × 512, and the batch size to 4. To achieve fast convergence when training the model, we used the Adam optimizer with an initial learning rate set at 0.001. The loss function in which we trained the network employed a combined cross-entropy loss and dice loss. All of our baselines were trained with the same settings and loss function.

## 5. Results

### 5.1. Comparative Experiment

The experimental results on the two datasets are shown in Table 1 and Table 2. As shown in Table 1, the UNet-based approach still has a good performance, with UNet++ and MRUNet outperforming MedT on GlaS. However, our approach is significantly superior to both the UNet-based and the Transformer-based methods.

**Figure 6:**
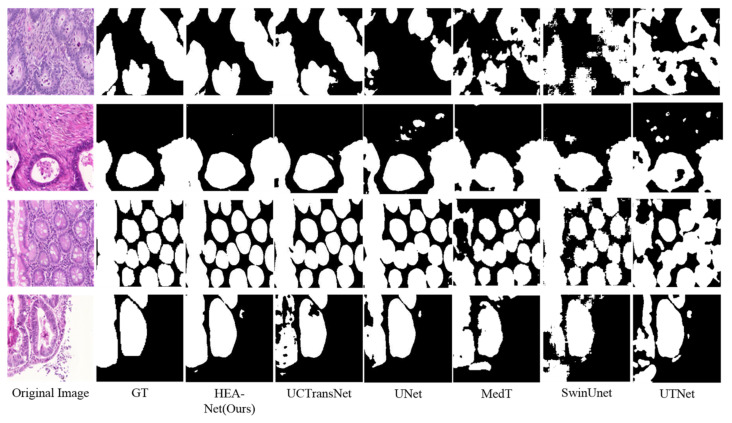
Qualitative results on GlaS dataset.

In the GlaS dataset, Dice and IoU reached 90.56% and 83.62%, respectively, obtaining the best Dice and IoU. We show the qualitative comparison results in Figure 6. It can be seen that: (1) UNet is more likely to cause over-segmentation or under-segmentation (for example, line 1, line 2, and line 4). (2) In UCTransNet, these phenomena are improved with Transformer. This shows that the Transformer-based hybrid model has strong global context modeling ability. However, due to Transformer’s weak local modeling ability, it can be seen from Swin-UNet’s forecast segmentation diagram that its segmentation contour is relatively rough. (3) Compared with other models, the segmentation effect of HEA-Net is better. Its segmentation profile is also smoother. It can accurately identify the edge of the lesion tissue (for example, the first and fourth lines).

**Figure 7:**
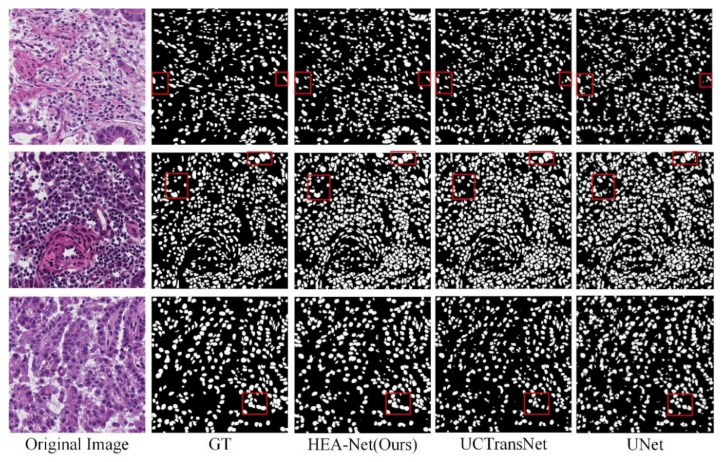
Qualitative results of MoNuSeg dataset based on UNet. In the red box are the parts with significant differences in the segmentation results of each network.

**Figure 8:**
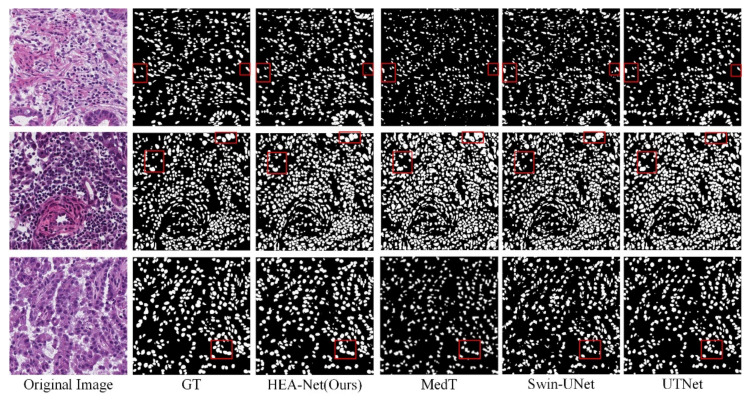
Qualitative results of MoNuSeg dataset based on Transformer. In the red box are the parts with significant differences in the segmentation results of each network.

In the MoNuSeg dataset, Dice and IoU reached 80.80% and 68.26%, respectively. As shown in Figure 7 and Figure 8, we offer the network segmentation results based on UNet and Transformer. It can be seen from the figure that: (1) UNet still has some cases of misclassifying background pixels as foreground pixels (for example, the first line of Figure 7). (2) The Transformer hybrid model ameliorates some of the incorrect predictions. However, Transformer’s poor sensitivity to local information is at a disadvantage for nuclear segmentation (see lines 1 and 3 in Figure 8). (3) Compared with other models, HEA-Net can capture the nucleus better.

### 5.2. Ablation Studies

To intuitively demonstrate the relative contribution of each module, we conducted ablation experiments on the GlaS and MoNuSeg datasets. We compared our model to the baseline (UCTransNet). As you can see from Table 3 and Table 4, both EAM and DS-MLP are indispensable, as removing either of them can result in network performance degradation. In addition, to demonstrate the critical role of the BN layer in the EAM module in our model, we included Baseline+EAM (without BN) in the ablation experiment. As shown in Table 3 and Table 4, the EAM with a BN layer performs better than the EAM without it. This is because the channel weights generated by the BN layer can highlight the importance of individual channels.

**Figure 9:**
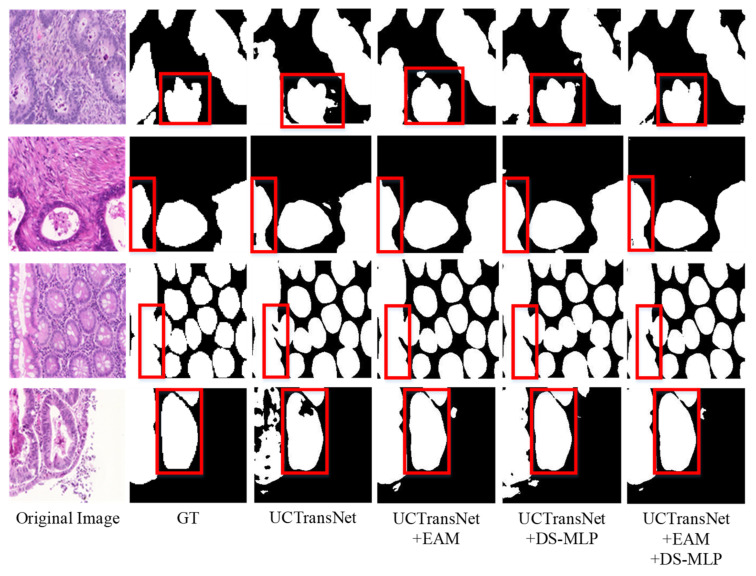
Visual comparison of each module in GlaS dataset. In the red box are the parts with significant differences in the segmentation results of each network.

On the GlaS dataset, Dice and IoU are 1.02% and 1.36% higher than the baseline (UC-TransNet), respectively. As shown in Figure 9, our model has remarkable performance in segmenting the edges of diseased tissue. The EAM module assigns unique weights to the pixel values from the channel dimension and spatial dimension, respectively, so as to strengthen the foreground information and weaken the background information. Taking the fourth row in Figure 9 as an example, the EAM module has a more accurate and smooth processing of the boundary of the foreground part, and the segmentation of the large areas is closer to the label graph (GT). DS-MLP simply further enhances the acquired features to avoid information loss. As you can see in the fourth row of Figure 9, the results with the addition of the DS-MLP module are significantly more complete than the baseline (UCTransNet), but slightly worse than the results with the addition of the EAM module. The combination of EAM and DS-MLP can better capture the foreground information of the samples. Therefore, HEA-Net has a solid ability to segment highly similar foreground and background information.

**Figure 10:**
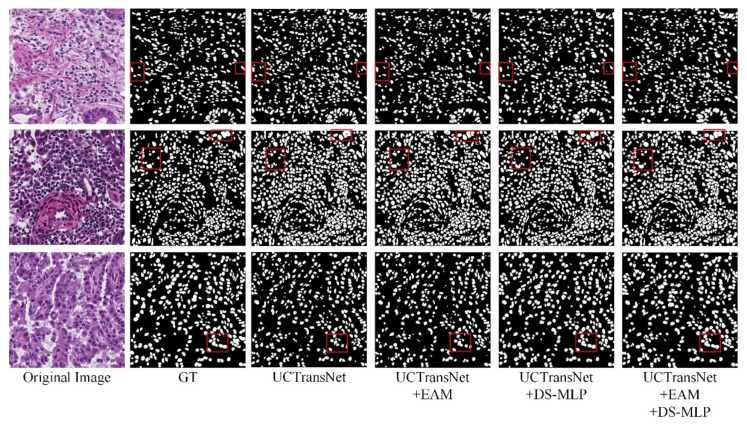
Visual comparison of each module in MoNuSeg dataset. In the red box are the parts with significant differences in the segmentation results of each network.

**Figure 11:**
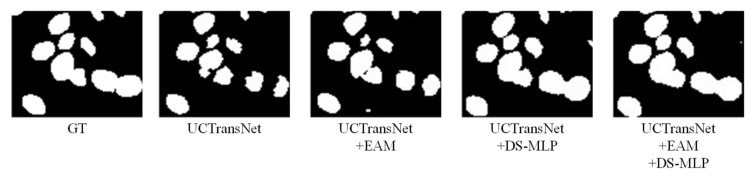
A local enlargement of the visualization result for the fourth line in Figure 10.

On the MoNuSeg dataset, Dice and IoU are 1.29% and 2.11% higher than the baseline (UCTransNet), respectively. As shown in Figure 10, most of the nucleus boundaries are closely connected, which increases the segmentation difficulty of our model. As can be seen from Figure 10, although the segmentation result of our model is improved compared to the baseline (UCTransNet), it is not very complete compared to the label map. From the fourth row in Figure 10, we derive a set of local enlargements (Figure 11). As shown in Figure 11, the segmentation graph with the DS-MLP module is more complete than that with the EAM module. This is because the DS-MLP module obtains salient features through hierarchical operation, which is better able to avoid the loss of important information. In order to correctly identify the boundary information of the nucleus, the network needs to pay more attention to the extraction of local details. The combination of EAM and DS-MLP can more accurately grasp the nuclear boundary information for local modeling. Thus, the HEA-Net segmentation of the nucleus is complete.

## 6. Conclusions

The accurate segmentation of medical images helps doctors to observe and judge the diseased parts of the human body more effectively. In this work, this paper proposes a hybrid attention encoder architecture (HEA-Net). In HEA-Net, we propose the EAM module can refine the convolutional output at different scales of the encoder network for better local modeling. The DS-MLP module is also proposed to achieve further feature enhancement by shifting operations in the channel and spatial dimensions. We evaluate HEA-Net on two public datasets. The experiments show that our network outperforms the most advanced methods. By analysis of ablation experiments on two different datasets, the overall performance of HEA-Net is significantly improved compared to baseline. Dice and IoU are (1.02%, 1.29%) and (1.36%, 2.11%) higher than baseline, respectively, indicating that the proposed HEA-Net, including EAM and DS-MLP modules, is helpful and valid for the segmentation of lesions.

## Figures and Tables

**Table 1:** Comparative experimental results on GlaS datasets. Network results with “*” are derived from [31]. The superscript “1” indicates the best experimental result.

Method	Dice (%)	IoU (%)
U-Net * (2015)	86.34	76.81
UNet++ * (2018)	87.07	78.10
AttUNet * (2018)	86.98	77.53
MRUNet * (2020)	87.72	79.39
UTNet (2021)	83.05	72.32
MedT * (2021)	86.68	77.50
Swin-Unet * (2021)	88.25	79.86
UCTransNet (2021)	89.54	82.26
HEA-Net (Ours)	90.56 ^1^	83.62 ^1^

**Table 2:** Comparative experimental results on MoNuSeg datasets. Network results with “*” are derived from [31]. The superscript “1” indicates the best experimental result.

Method	Dice (%)	IoU (%)
U-Net * (2015)	73.97	59.42
UNet++ * (2018)	75.28	60.89
AttUNet * (2018)	76.20	62.64
MRUNet * (2020)	77.54	63.80
UTNet (2021)	78.35	64.74
MedT * (2021)	79.24	65.73
Swin-Unet * (2021)	78.49	64.72
UCTransNet (2021)	79.51	66.15
HEA-Net (Ours)	80.80 ^1^	68.26 ^1^

**Table 3:** Ablation experiments on GlaS datasets. The superscript “1” indicates the best experimental result.

Method	Dice (%)	IoU (%)
Baseline (UCTransNet)	89.54	82.26
Baseline + EAM (without BN)	87.72	79.51
Baseline + EAM	89.86	82.48
Baseline + DS-MLP	90.20	83.04
Baseline + EAM + DS-MLP (Ours)	90.56 ^1^	83.62 ^1^

**Table 4:** Ablation experiments on MoNuSeg datasets. The superscript “1” indicates the best experimental result.

Method	Dice (%)	IoU (%)
Baseline (UCTransNet)	79.51	66.15
Baseline + EAM (without BN)	77.99	64.82
Baseline + EAM	80.20	67.57
Baseline + DS-MLP	80.41	67.51
Baseline + EAM + DS-MLP (Ours)	80.80 ^1^	68.26 ^1^

## Data Availability

The GlaS and MoNuSeg datasets are openly available at: https://warwick.ac.uk/fac/cross_fac/tia/data/glascontest (accessed on 8 July 2022) and https://monuseg.grand-challenge.org/Data/ (accessed on 8 July 2022).
